# Preparation and Characterization of a Type of Green Vacuum Insulation Panel Prepared with Straw Core Material

**DOI:** 10.3390/ma13204604

**Published:** 2020-10-16

**Authors:** Lu Wang, Yong Yang, Zhaofeng Chen, Yiyou Hong, Zhou Chen, Jiankun Wu

**Affiliations:** 1 Department of Flat Panel Display, Nanjing Electronic Devices Institute, Nanjing 210016, China; Venuswang0915@126.com (L.W.); Razernaga@126.com (Y.H.); 2 National Engineering Laboratory for Modern Silk, College of Textile and Clothing Engineering, Soochow University, Suzhou 215000, China; 3 College of Material Science and Technology, Nanjing University of Aeronautics and Astronautics, Nanjing 210016, China; zhaofeng_chen@163.com; 4 School of Mechanical and Power Engineering, Nanjing Tech University, Nanjing 211800, China; zchen6240@njtech.edu.cn; 5 Research and Development Department, Suzhou Zhenlun Spinning Co., Ltd., Suzhou 215000, China; welcome213441@126.com

**Keywords:** straw VIP, moisture content, compression strength, thermal conductivity, drying process

## Abstract

The Vacuum Insulation Panel (VIP), regarded as the most promising high-performance thermal insulation material, still has application limitations because of its high cost. In this paper, VIPs using natural straw as the core material are prepared. The fiber saturation point (FSP) is important in order to determine the optimum for the use of renewable straw materials as a potential VIP core. The microstructure of straw core material, together with the relationship between the moisture content, the diametral compression strength, and the thermal conductivity of as-prepared straw VIPs are investigated. Compression characteristics of straw core material and heat insulation mechanism within the straw VIP envelope enclosure are analyzed. Total thermal conductivity of a straw VIP is sensitive to both the inner pressure and the moisture content of straw core material. The optimum drying process for straw VIPs is heating the straw core material at a temperature of 120 ℃ for 60 min, with its center-of-panel value being about 3.8 mW/(m·K).

## 1. Introduction

Vacuum insulation panels (VIPs) represent outstanding thermal insulation performance with a thermal resistance of about 10 times larger than that of equally thick conventional insulation materials and could make a great contribution to energy saving in buildings [1].

A VIP is basically made of a micro-porous core structure which is evacuated and sealed in a thin, virtually gas-tight envelope bag [2]. Fricke et al. stated that the core material had to be porous to easily evacuate and to have a minimum solid conduction effect. For this reason, bulk type materials in the form of powders, foams and fibers have been frequently used as the core material [3]. The polystyrene (PS) foam, polyurethane (PU) foam and phenolic foam (PF) as the core materials were investigated in [4,5,6]. Powders have both high compression strength and thermal conductivity; insulation foams have been used since the early stage thanks to their low price; however, they have relatively poor insulation performance and large pore size, and fibers have good thermal resistance but poor structure stability [7,8]. 

Jae-Sung Kwon et al. stated that foam is not suitable for use as a core material in practical applications because of its high solid skeleton thermal conduction [9]. Silica aerogels were discovered in the early 1930s by Kistler. In general, aerogels had a high specific surface area, a very low apparent density, a low refraction index and super thermal resistance, less than 20 mW/(m·K) at ambient conditions [10]. VIPs made of glass fibers were usually used in high-temperature applications due to their low density and high thermal stability. Nevertheless, conventional VIPs cost a little bit more because of the relatively expensive raw materials and complex preparation technical processes, which to some extent limit the wider application of VIPs as thermal insulators around the nation. Considering that natural straw fibers are mass produced each year as a renewable abundant natural fiber [11] and some straw buildings have been built due to the straw’s good combination of eco-sustainability, low cost and energy efficiency [12], it could be more environmentally friendly and economically beneficial to produce green VIPs using straw fibers as core material.

This paper, based on the requirement of green building energy saving, researched the preparation, properties and mechanism of VIP which utilized renewable resources, namely, straw, as its core material. The aim of this paper was to determine the best drying process of straw core material and obtain a new kind of high-performance green VIPs.

## 2. Experimental Procedure

### 2.1. Materials

Raw materials of the wheat straw (straw for short hereinafter) used in this study was provided by the Council of Agriculture in Shiqiao town, Lianyungang (Jiangsu, China). Raw materials of the getter and envelope materials used in this study was provided by Suzhou V.I.P. New Material Co., Ltd. (Taicang, China). Two kinds of hybrid envelopes were used as VIP barrier envelope materials in this manuscript. One is the laminated aluminum foil (AF) which is made up of four-layered films, i.e., 15 μm polyamide (PA) film, 12.1 μm aluminum-polyethylene terephthalate (Al-PET) film, 6.3 μm aluminum (Al) film and 50 μm polyethylene (PE) film. The other is the multilayer membranes with metalized film (MF) which is made up of three-layered films, i.e., 12.1 μm Al-PET film, 12.1 μm Al-PET film and 50 μm PE film.

### 2.2. VIP Fabrication

#### 2.2.1. Core Material

The wheat straw core materials were prepared via three steps. Firstly, the natural wheat straw was cut into short straw of 50 mm to 150 mm long by a low frequency cutting machine. Then the leaves, spikelet, and impurities were removed by an air-separator. Finally, it was ensured that the lengths of the short wheat straw were 5 mm to 60 mm after being cut a second time.

#### 2.2.2. Getter

About 8–12 g of CaO powder was used as desiccant and 2–6 g of wheat husks was burnt to ash as getter. Each of them was in an individual package.

#### 2.2.3. VIP

**Figure 1:**
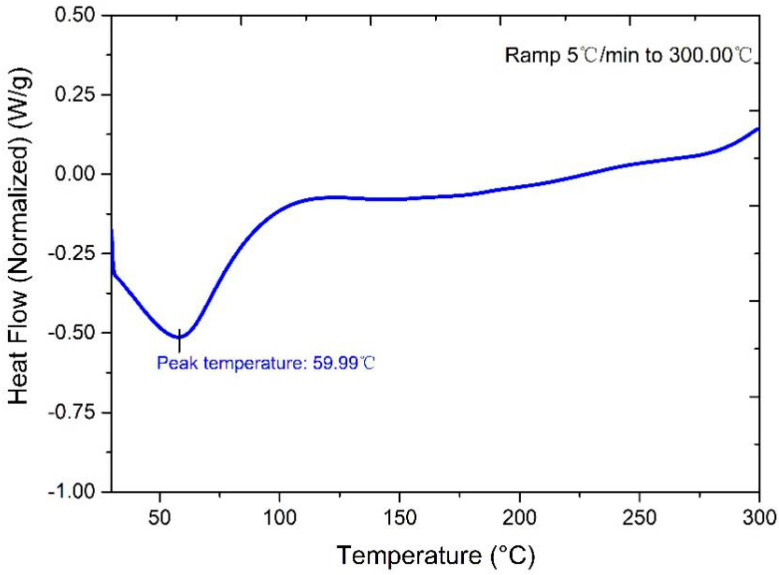
Differential scanning calorimetry (DSC) graph of straw core material.

It is well known that heat treatment of fibrous materials can speed up the motion of molecules in the pores and accelerate the outgassing. The moisture and volatile substances attached at the surface of fibers can be transformed into superheated gases and then located in the pore space after high temperature heat treatment up to 180 °C. Therefore, the drying process eased subsequent vacuum processes. According to the research in [13], the straw was easy to carbonize because of its biologic characterization. It can be seen from Figure 1 that there is an exothermic peak at approximately 60 °C, which means the free water on straw walls and lumens starts evaporating.

The whole evaporating process does not end until the heating temperature reaches about 120 °C, which means there is nearly no free water left at 120 °C. The bound water loss in straw cells begins to follow the rising temperature, which is an endothermic process. The Differential Scanning Calorimetry (DSC) curve does not fluctuate obviously at 180 °C, so there are no chemical reactions taking place at 180 °C.

**Figure 2:**
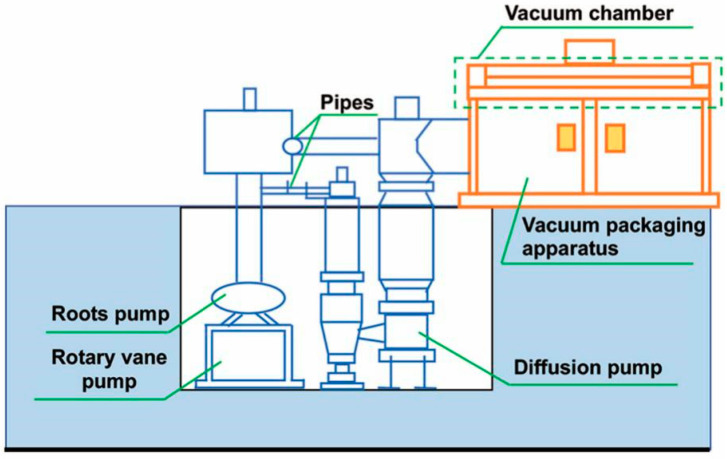
Schematic drawing of vacuum equipment [14].

Hence, in order to get the intact straw core materials for moisture content analysis, the drying temperature was set to 60 °C, 90 °C, 120 °C, 150 °C and 180 °C respectively for 60 min. At first, the core materials were distributed into 6 parts, one part was not dealt with, and the other parts were dried at 60 °C, 90 °C, 120 °C, 150 °C, and 180 °C respectively for 60 min. Then all core materials were bagged in six envelope bags. Afterwards, the six groups of straw VIPs were produced by an evacuation process step followed by the sealing of the envelope and the air bleeding of the vacuum chamber (see Figure 2). 

**Figure 3:**
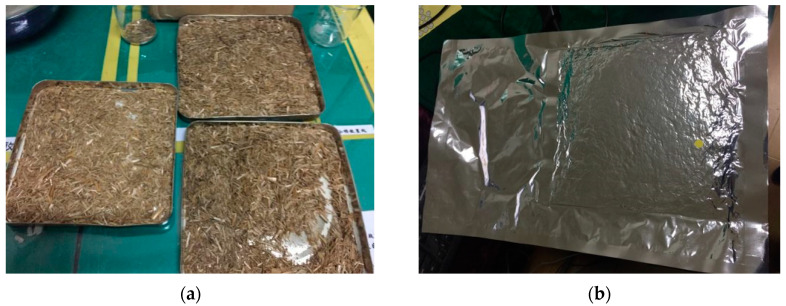
Photos of straw core material after drying process (**a**) and prepared straw Vacuum Insulation Panel (VIP) (**b**).

Gases within the vacuum chamber can be evacuated by the synergistic work of rotary vane pump, roots pump, and diffusion pump, which ensured the extraction process proceeded sequentially to a high-quality vacuum level. Envelopes were heat sealed after a pressure holding process of 5–10 min. Eventually, the vacuum chamber was vented and the straw VIPs were prepared (see Figure 3).

### 2.3. Characterization

The morphology of straw core material was observed by scanning electron microscopy (SEM, SU8010, Tokyo, Japan). Samples were treated by spray gold process (HM3260) before SEM test. The element of straw core material was tested by Energy Disperse Spectroscopy (EDS, X-Max 50, Oxford, UK). The moisture content of straw core materials was tested by differential scanning calorimetry (DSC Q20, DE, USA) according to ISO 11357-1. The diametral compression strength of straw core materials was recorded by SANS Universal Testing Machine. The inner pressure of VIP was tested by a system containing Spinning Rotor Gauge (SRG, Tel Aviv, Israel), as well as extra an opening for VIP evacuation which was supplied by Yoash Carmi et al. [15]. The thermal conductivities of as-prepared straw VIPs were evaluated by heat flow meter (Netzsch HFM 436, Selb, Germany) in accordance with ISO 8301 which represents the center-of-panel value of such VIPs. The accuracy of the heat flow meter (Netzsch HFM 436) is 1–3%. The detailed pore diameter was calculated from SEM pictures. For every sample, at least 50 holes in SEM pictures were measured. The average value was calculated.

**Figure 4:**
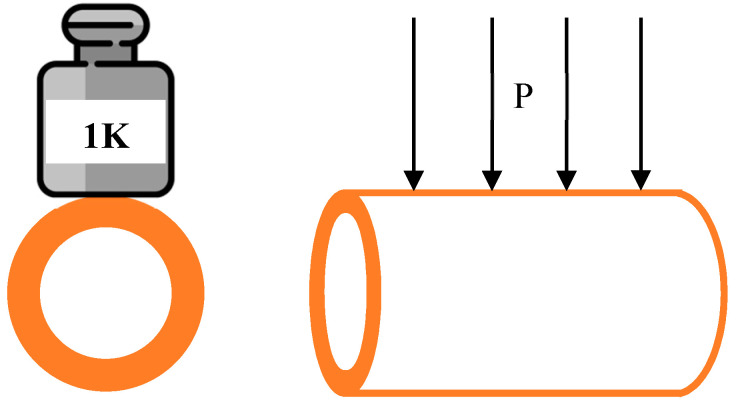
Testing method for the compression capability of a single straw.

We used two methods to characterize the compression characteristics, also called the radial compression stiffness. To test the maximum radial compression stiffness, we utilized the same method as the method reported in [16]. However, we also wanted to test the compression capability of a single straw. Therefore, we used a simple method by putting a 1 kg weight on single straw for 1 min as shown in Figure 4, observing the differences of the cross-section view among those samples.

## 3. Results and Discussion

### 3.1. Material Characterization

**Figure 5:**
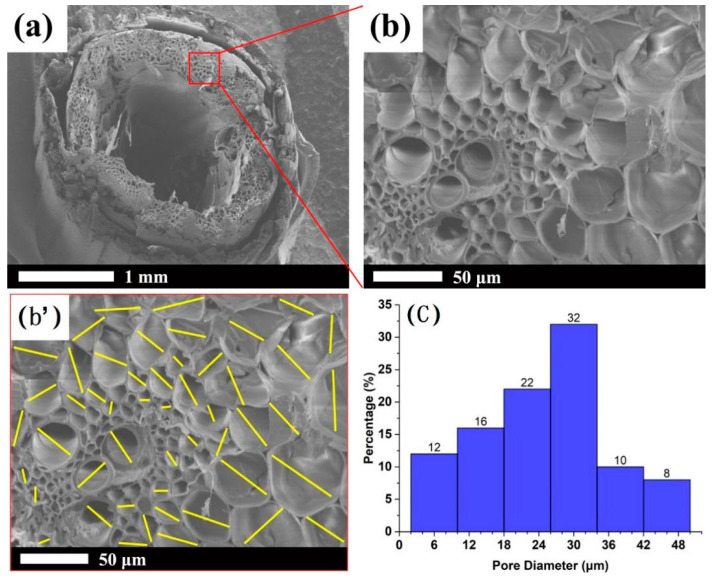
SEM micrograph of the straw core material: cross-section of single straw (**a**), partial enlarged detail (**b**), sampling tags of pore size for straw core (**b’**) and pore size distribution graph (**c**).

Jae-Sung Kwon et al. proposed that the flat type VIP must have a core material which withstands the atmospheric pressure and a gas-tight envelope maintaining the inside vacuum level. The core material had to be porous to be easily evacuated, and to have minimum conduction heat transfer effect [9]. Figure 5 shows the SEM micrograph of the straw core material.

The straw has an approximate 1 mm hole in the middle. According to the pore size distribution graph of straw core material in Figure 5c, the straw wall is made up of pores with size range between 2–50 μm. Thus, straw is a material with macropore and micropore structure, and this contributes to much higher porosity and a much smaller pore diameter. The multi-layered porous structure helps to limit the movement of gas molecular, consequently reducing the heat conduction and the heat convection [17].

At present, core materials for VIP include but are not limited to PU, PF and PS. Table 1 gives the main performance parameter comparisons of VIPs prepared with the above-mentioned core materials.

It could be found that VIPs prepared with different core materials all possessed a high porosity of over 93%. Among all the core materials, VIP made of a straw core had the smallest pore diameter and the lowest thermal conductivity. Furthermore, thanks to the renewable, green eco-friendly and low-cost straw natural resources, futural VIP core material tends to be straw.

The average bulk density is about 100 kg/m^3^. The straw fiber diameter is 0.1–1.3 mm. The pore diameter is 2–50 μm. The porosity of the straw core material is no less than 95%. The size of squared VIP specimens is 30 × 30 × 1 (cm^3^).

### 3.2. Thermal Insulation Mechanism

The thermal conductivity is regarded as the most important property of a thermal insulation material. The total overall thermal conductivity λ_tot_ of VIPs was made up from several contributions in principle [14,18], shown in Equation (1):

λ_tot_ = λ_solid_ + λ_gas_ + λ_conv_ + λ_rad_ (1)

where λ_solid_ is solid thermal conductivity, λ_gas_ is gas thermal conductivity, λ_conv_ is convection thermal conductivity, and λ_rad_ is radiation thermal conductivity. λ_rad_ was less important when measuring at ambient temperature but dramatically increases with temperature [19] and λ_conv_ should be negligible as the mean straw pore size was far below 1mm [20,21]. λ_solid_, associated with thermal transport between atoms by lattice vibrations, and λ_gas_, linked with gas molecules colliding with each other, were both supposed to be reduced to a maximum state to achieve VIPs of high thermal insulation performance, which were closely related to the pore size and porosity of the core material [22].

**Figure 6:**
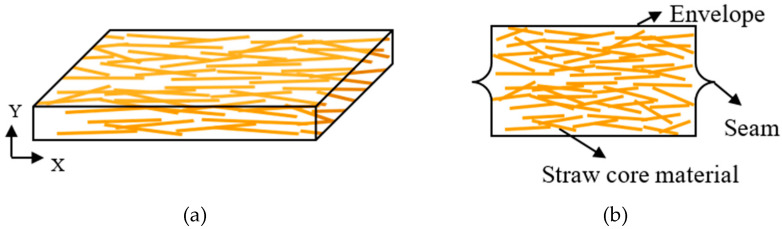
Schematic diagram of VIP with straw core material: 3D schematic diagram of VIP (**a**) and cross section of VIP (**b**).

Moreover, a low λ_solid_ needs high porosity and low density while a low λ_gas_ is related to the tiny pore size of the core material and the low vacuum degree. Obviously, the straw core material was bearing near an atmospheric pressure after the vacuum process, which relied on its good compression strength to withstand the pressure. The characteristics of core material, such as porosity, and density, were determined. Figure 6 shows the schematic diagram of VIP with straw core material.

The straw core material has a horizontal distribution in the direction of thickness, layer by layer, with the straw fibers staying oriented and uniform in the plane of the side view. Consequently, radial forces were applied on each straw unit after the vacuum process, as shown in Figure 6b. In addition, a single straw unit has the shape of a hollow circular cylinder with a large void in the center, which seems to be a shortcut for gas conduction. However, the straw core material is multilayered like glass fiber felt so that the free gas conduction through the fiber void only happens in the plane orientation, rather than the vertical direction of VIP. Thus, the void in center of the straw does not have an efficient effect on the gas heat conduction across the panel.

Cheng-Dong Li et al. stated that the real morphology of glass fiber core material can be described by a multiphase medium model. Representative elemental volume in this model at microscopic level consists of solid phase, gas phase, and impurity phase. Gas phase filled in the voids among solid phases while the impurity phase attached to the surface of the solid phase, which might change with the increasing temperature [23].

**Figure 7:**
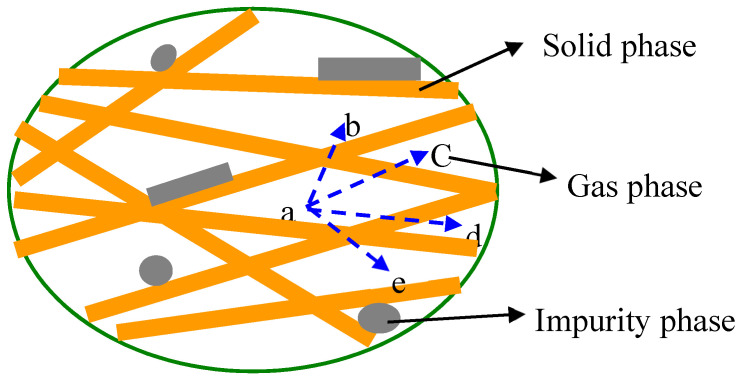
Multiphase medium model for straw core material [23].

**Figure 8:**
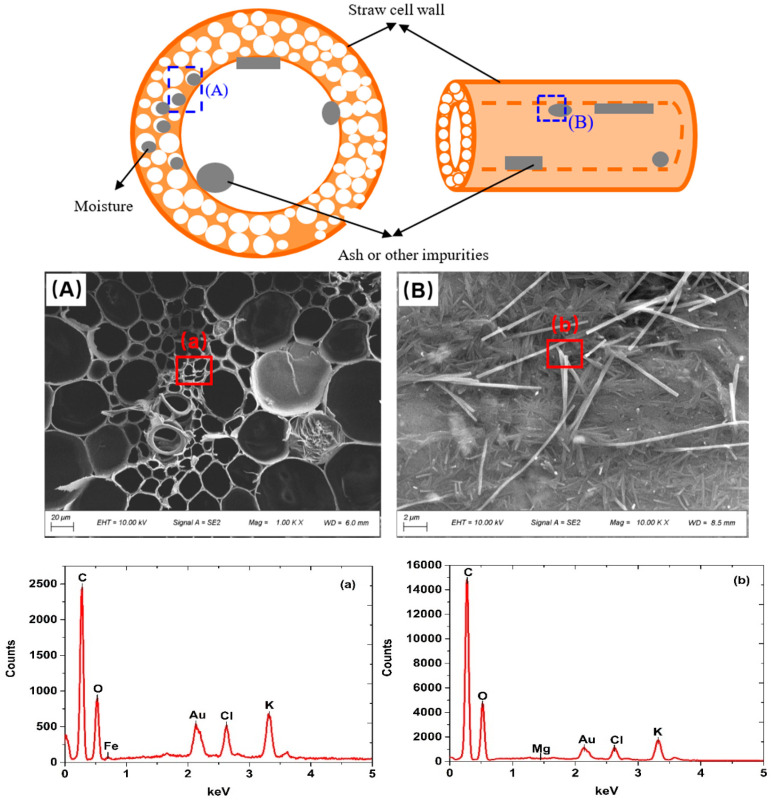
Schematic diagram and SEM/EDS photos of a single straw unit: cross-section (**A**) and straw cell wall (**B**). (**a**) elements tested from part (**A**), (**b**) elements tested from part (**B**).

Figure 7 shows the multiphase medium in core material and Figure 8 shows the schematic diagram and SEM/EDS of a single straw unit.

The SEM photo in Figure 8, which was consistent with Figure 5, proved that the microstructure of the model in Figure 8A is reasonable. Signals in EDS photos showed that there were similar ratios of carbon to oxygen both on the cross-section and the cell wall of straw unit. This means that components in the cross-section and the cell wall of straw unit are similar, i.e., the straw sample homogeneity is good. The presence of Au signal is due to the normal process of spray gold treatment before the SEM test. It can be seen from EDS photos that content like Cl, K, Fe and Mg is low, which is in agreement with the research results in reference [24]. 

Then from the SEM photos, we can see that straw fibers have plenty of space to store moisture, ash or impurities thanks to their natural micro porous structure. Water vapor mainly transfers through the air spaces and is associated greatly with the capillary properties of core material. The straw cells consist of plenty of cellulose, which means the moisture is easy to absorb on the straw cells. The internal moisture preferred to flow towards the pores and occupy the pore space until the inner pressure achieved a balance in the end. Meanwhile, the existence of ash in the single straw unit would also change the microstructure of the straw unit which would further affect the thermal insulation performance of the core materials. 

### 3.3. Thermal Conductivity, Diametral Compression Strength and Moisture Content Analysis

**Figure 9:**
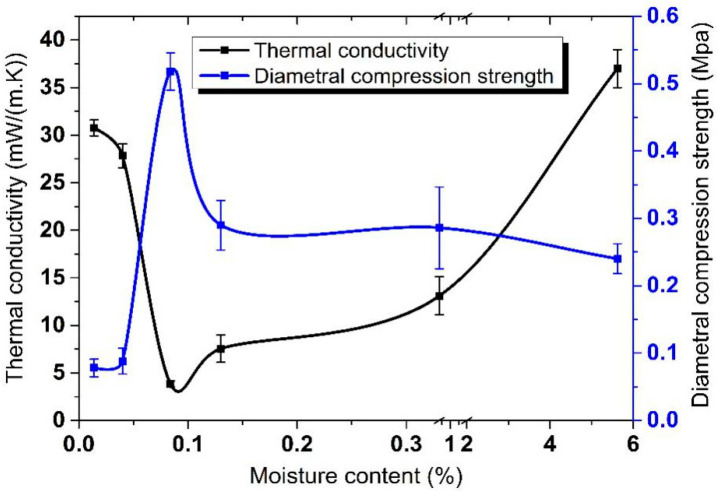
Relationship between thermal conductivity, diametral compression strength and moisture content.

Thermal conductivity, diametral compression strength and moisture content are pairwise correlated. The drying temperature of the core material is a significant parameter. Table 2 gives various properties at different drying temperature, generating the graph in Figure 9.

The standard deviation (σ) and the standard error (SE) of thermal conductivity and diametral compression strength were calculated according to Equation (2),

(2) $$\sigma =\sqrt{\frac{\Sigma {\left(X-\mu \right)}^{2}}{N}},\mathrm{SE}=\frac{\sigma }{\sqrt{N}}$$

where σ is the standard deviation, X is the tested value, μ is the average value, N is the number of population, SE is the standard error.

According to Equation (1) and Figure 9, thermal conductivity of the straw VIP mainly relies on heat conduction (λ_solid_) and heat convection (λ_gas_) of performance of the core material.

**Figure 10:**
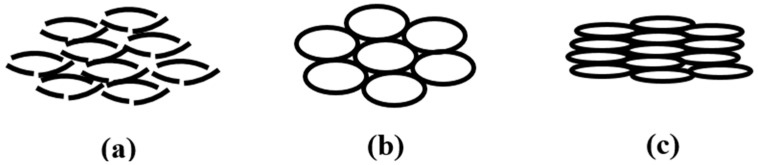
Different compression conditions of straw core material: collapse (**a**), intact (**b**), and deformation (**c**).

The blow figure shows the changes of core material in different conditions. With the increase of the moisture in the straw, the brightness of the straws decreases and their corresponding toughness increases. This means that the straw with the lowest moisture (0.01%) possesses the maximum brightness whose microstructure is easy to destroy with external pressure, as shown in Figure 10a.

The straw with the maximum moisture (5.62%) possesses the maximum toughness, and its microstructure is easy to flatten with external pressure, as shown in Figure 10c. In the above two situations, the solid content is obviously increased under the same thickness, and their solid heat conduction is increased. For the situation of Figure 10b, the straw possesses the maximum compression to resist the external pressure because of the relatively low moisture (0.08%) content and appropriate toughness. In addition, the increase of the moisture leads to the continuous increase of the heat convection. Therefore, the sample with 0.08% moisture possesses the minimum thermal conductivity. The thermal conductivity of samples increases with the increase of the moisture.

**Figure 11:**
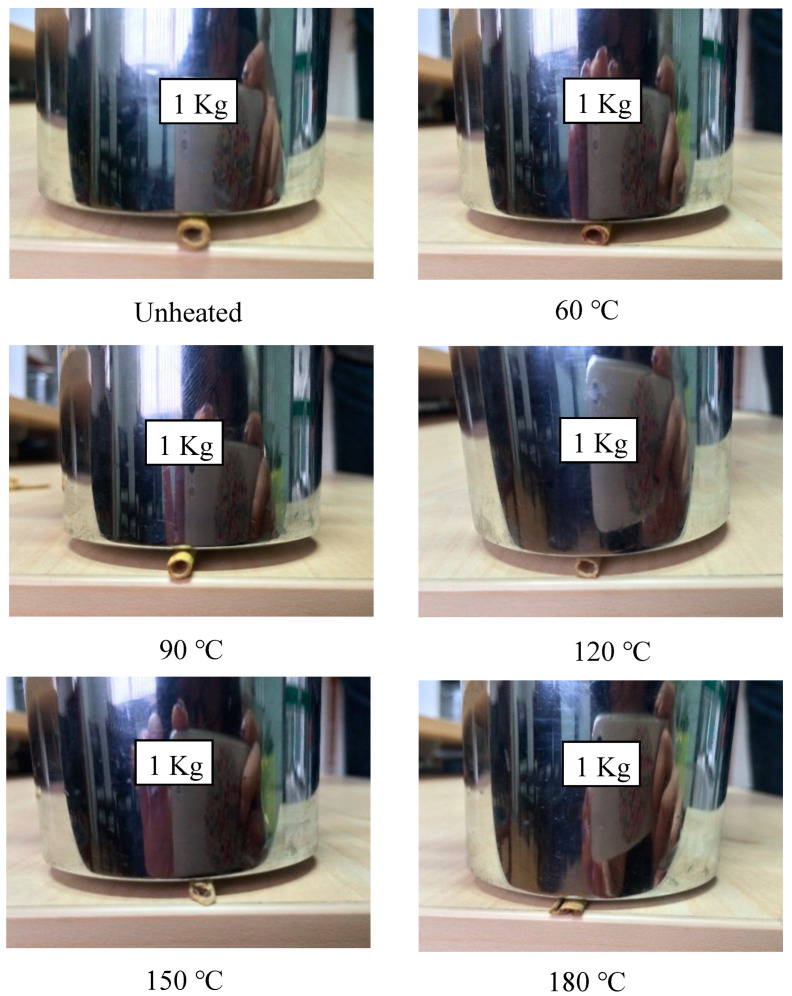
Real photos of straw units drying at different drying temperatures under the approximate pressure of 1 bar.

Figure 11 shows the real photos of straw units at different drying temperatures presenting different cross-section shapes on the same force situation.

Straw units heated at 60 °C, 90 °C, and 120 °C remained, like the unheated one, unbroken. The one heated at 150 °C appeared to crack and the one heated at 180 °C totally collapsed. It was obvious to see from Table 2 that only the two groups of straw units heated at 150 °C and 180 °C had the diametral compression strength lower than 0.1 MPa, so that those two specimens were broken while the others remained intact. The worst structure collapse of straw appeared after being treated at 180 °C, so its thermal conductivity is higher than that treated at 150 °C. As a result of the structure destruction of straw core material treated both at 150 °C and 180 °C, their thermal conductivities were apparently higher than the other samples treated at lower temperatures.

To explain the relationship between thermal conductivity, diametral compression strength and moisture content (see Figure 9), it is necessary to determine the Fiber Saturation Point (FSP). It was stated by Eitelberger et al. that FSP was the moisture content at which the cell walls were saturated with bound water, leaving the cell lumens still empty, which is considered to be crucially important to the effect of compression [25]. Generally, different wood species have different FSP. Conditions below FSP are the normal state of wood when used for structural purposes [26]. In this article, Moisture in straw consists of free water and bound water. During the process of heat treatment, free water is firstly removed. Then, bound water, which plays an important role in structure strength of straw core material, will not be destroyed until there is no free water left. On one hand, too much free water contributes to higher gas thermal conductivity thanks to water vapor heat conduction in the Vacuum. Too little bound water will lead to higher solid thermal conductivity because of the decline of compression capability. When the moisture content of straw was above the FSP, bound water and free water all existed in the straw cell walls and lumens. At the moisture content of 5.62% where free water occupied a high proportion of the whole moisture content, the increased capacity of free water diffusion due to the differential concentration and the good fluidity of particles accounted for the low diametral compression strength of straw core materials. Lower diametral compression strength leads to higher solid heat conduction. The temperature point at which free water was completely removed and bound water was well maintained was 120 °C. That is to say, core material treated at 120 °C has the lowest gas thermal conductivity and lowest solid thermal conductivity so that thermal conductivity at 120 °C was the lowest.

According to the fibrous insulation model reported by Jae-Sung Kwon et al. [27], where k_f,fiber_, the effective thermal resistance of solid fiber core materials, can be derived as Equation (3):

(3) $$k_{s,\mathrm{fiber}}{=16k}_{f}[(\frac{\sqrt{2}\pi^{4}E}{{24P(1-\Pi }^{4}{)(1-\upsilon }^{2})}{)}^{1/3}+\frac{\pi^{2}}{{4(1-\Pi )}^{3}\sin^{2}\theta }{]}^{-1}$$

Of all the parameters in Equation (3), solid conductivity of straw fiber k_f_, Young’s modulus of elasticity E, and pressure P are constant. Supposing the Poisson’s ratio ν and tilted angle of fibers θ stay the same, only the porosity Π would change with the compressive capacity of straw core materials. k_f, fiber_ is inversely proportion to Π. Kim et al. [5] also stated that solid conductivity can be estimated using the porosity of the core material that has non-uniform microstructures. Overall, for straw core materials of the same original quality, the lower the diametral compression strength of straw core material is, the more easily the straw fibers will collapse, which decreases the porosity of straw core. Thus, the k_f, fiber_ of straw core increases.

**Figure 12:**
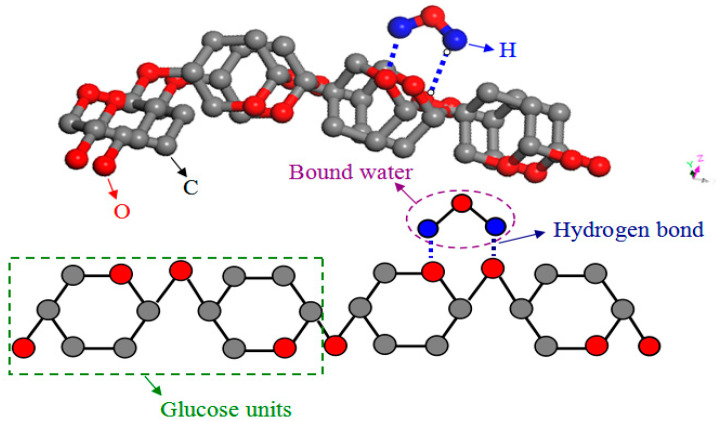
Hydrogen bond formation between bound water and the amorphous region of the cell.

In this case, not only λ_solid_ but also λ_gas_ was increased because the water vapor within the VIP envelope enclosure coming from the high amounts of interparticle moisture was increased, resulting in the highest total thermal conductivity of straw VIP. With the reduction of free water, the moisture content arrived at 0.34% and 0.13%. The squeezed straw particles deformed and thinned the cell vessels which added resistance of the water transport. Meanwhile, the lower proportion and damping diffusion capacity of free water enhanced the extension resistance of straw particles, making the compression strength relatively high. Thus, the relative reduction of λ_solid_ and λ_gas_ led to a lower total thermal conductivity of straw VIP. It was explained by Habets et al. that wheat straw was mainly made up of cell wall components hemicellulose, cellulose and lignin, also including significant amounts of inorganic material (ash) and extractives. Moreover, cellulose, as the most prevalent cell wall component in lignocellulosic biomass, was known to exist in the amorphous region as well. Linear polysaccharide constructed of thousands of glucose units can align alongside each other through hydrogen bond, resulting in a highly crystalline substance [28]. When the moisture content of straw was below the FSP, only bound water was left, and it was easy to form a hydrogen bond between the bound water and the amorphous region of the cell wall (see Figure 12).

Such intermolecular forces were the main contributors to resisting pressure distortion. At the moisture content of 0.04% and 0.01%, the bound water was massively removed, causing a great reduction of the compression strength and complete collapse of straw core material when stressed. Thus, the λ_solid_ of straw core material increased a lot. 

**Figure 13:**
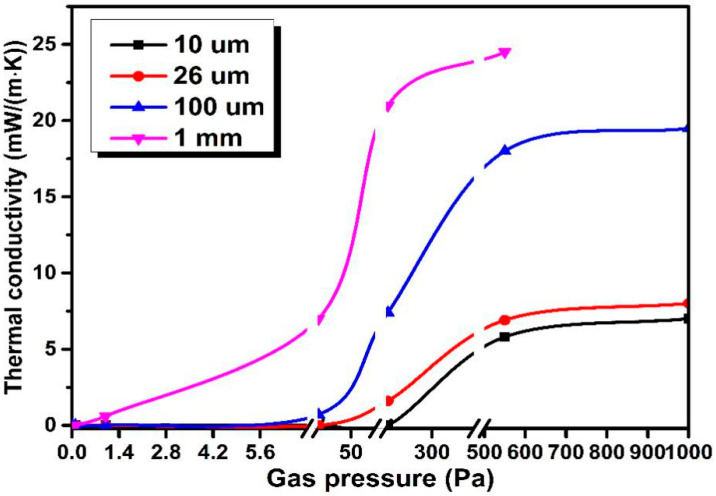
Gaseous conductivity with different pore sizes as a function of gas pressure.

On the other hand, Kwon et al. reported that ideal pore size would be 10 μm, at which the gas conductivity would be nearly zero at a pressure of 10 Pa. They showed how gaseous conductivity changed with different pore sizes as a function of gas pressure (see Figure 13) [27].

As for the straw core materials, there are two kinds of pores. One is the vascular bundles of varying pore sizes inside the straw fiber due to the characteristics of natural plant fiber. The other constitutes large and small gaps as a result of mutual cross lap joints of straw fibers. Therefore, the straw core material has a relatively high porosity and a pore diameter span ranging from 2 μm to 50 μm (mean 26 μm) just because of the heterogeneity of straw fiber pore size. Observing the curve trend from Figure 13, it can be predicted that the gaseous conductivity with pore size of 26 μm would still stay at a lower level if the gas pressure was below 100 Pa, which means the pore size of straw core material might be a good choice for air molecules to collide with the pore surface without transferring energy by elastic impact. With the vacuum degree reaching appropriate value, the straw VIP could achieve a low λ_gas_. Certainly, VIPs with straw core material are more sensitive to pressure increases in vacuum compared with glass fiber core or fumed silica core materials because the latter pore size [29,30] is smaller than that of straw core material. Jae-Sung Kwon et al. [27] also reported that the gaseous thermal conductivity k_g_ can be derived as Equation (4):

(4) $$k_{g}=\frac{k_{g_{0}}}{1+\frac{0.032}{P\Phi }}$$

where P is the inner pressure of VIP, Φ is the pore size of core material. Fricke et al. [3] stated that VIPs with glass core material and fumed silica core material respectively showed a doubling of conductivity, once the gas pressure surpassed 100 Pa and 10,000 Pa. Thus, for the glass fiber core with pore diameter of 8 μm, the calculated gaseous conductivity at the inner pressure of 100 Pa is 6.34 × 10^−4^ W/(m·K). For the straw core with average pore diameter of 26 μm, the inner pressure only needs to be 30.77 Pa to reach the gaseous conductivity level of 6.34 × 10^−4^ W/(m·K).

Because of the degradation of the core material, the service life of straw VIP is supposed to be much shorter than glass fiber VIP or fumed silica VIP. In order to prolong its service life in future research, some solutions have to be figured out, for example, using more appropriate getter and desiccant, adopting envelopes of better barrier properties to maintain the vacuum level inside the straw VIP for a longer time, and even mixing the straw core with fumed silica particles or glass fibers to decrease the pore size of the composite core materials. 

Vacuum level within the VIP envelope enclosure changing with time was recorded in Table 3.

Straw VIPs with 2 amounts of desiccant and getter mixtures are compared with each other. A represents VIP with 12 g of CaO powder desiccant and 2 g of wheat husks ash getter and B represents VIP with 8 g of CaO powder desiccant and 6 g of wheat husks ash getter. Overall, the two amounts of desiccant and getter mixtures work similarly on the absorption of gases and water vapor. To some extent, mixture B performs a little better than mixture A. These six groups of straw VIP samples’ inner pressure data are recorded after 3 days, one week, and one month respectively. It turns out that the increase of inner pressure is small and finite in the test duration of one month, which indicates that a good vacuum level is still maintained within the enclosure of VIP samples after one month. Therefore, the vacuum level could be held for at least one month or even longer. That is to say, λ_gas_ caused by gases and water vapor could be ignored. Even so, the λ_solid_ of straw core material grew a lot so that the total thermal conductivity of straw VIP was raised correspondingly. Therefore, a system of checks and balances exists. At the moisture content of 0.08%, namely around the FSP, the total thermal conductivity of straw VIP was the lowest with both of the solid and gas heat conduction acting synergistically.

**Figure 14:**
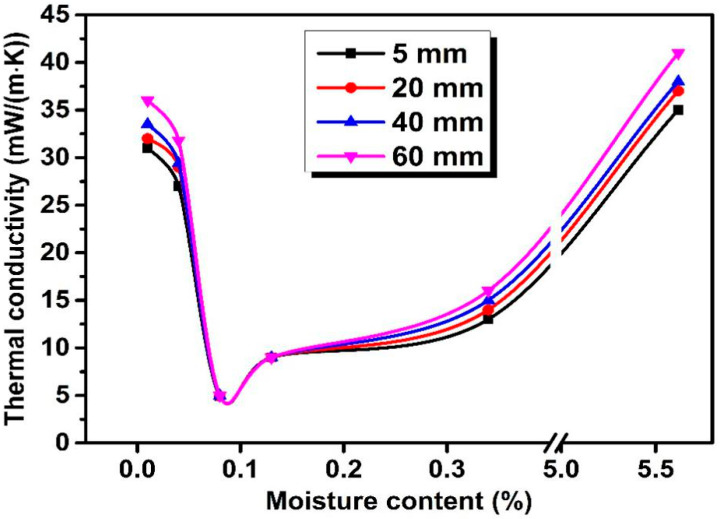
How thermal conductivities of straw VIPs made of different core fiber length change with moisture content of straw core.

Figure 14 shows the relationship between the moisture content of straw core material and thermal conductivities of straw VIPs of different fiber length.

The λ of VIPs possessing straw length of 5 mm, 20 mm, 40 mm, 60 mm was marked as λ_5mm_, λ_20mm_, λ_40mm_, λ_60mm_, respectively. All the four types of VIPs were prepared at the same condition. The initial λ of VIPs at the moisture content of 5.62% was lower than 45 mW/(m·K) and decreased gradually following the reduction of moisture content until the value of moisture content reached 0.08%. However, a sudden increase of the λ of VIPs appeared as the moisture content decreased further from 0.08% to 0.01% during the drying process. This phenomenon might be closely connected with the diametral compression strength of straw core materials, which will be explained below.

On the other hand, the λ of VIPs rose slightly with the straw length while the density of straw core material remained the same, i.e., λ_5 mm_ < λ_20 mm_ < λ_40 mm_ < λ_60 mm_. This could be proved by a 3D model of random cellulosic fibrous networks established by M. Faessel et al., which presented the parameters of the model corresponding to its internal architecture properties and studied their influence on a local thermal conductivity. While it turned out that the length and the horizontal orientation of the fibers seemed to have a very low influence (less than 10%) on the network thermal conductivity compared with other parameters such as the density, the vertical orientation and the tortuosity of the fibers [31]. This is mainly because of the same density of straw core material, which is the direct reflection of porosity. All the four groups of straw VIPs have nearly the same λ_solid_. Apparently, each straw fiber unit is rigid, which is different from other flexible fibers. Thus, the four straw VIPs have the same tortuosity which turns out to be 1, according to the definition of tortuosity—the ratio between the length of the fiber and its generating line—which was explained in reference [31]. Additionally, the distribution of straw fibers is disorganized both horizontally and vertically so that the four straw VIPs share almost the same influence that both the horizontal and vertical orientation of straw fibers have on the network thermal conductivity. Thus, the thermal conductivities of those four straw VIPs differ slightly from each other because of the fiber length differences. It was pointed out in reference [31] that shorter fibers had lower thermal conductivity. To explain this theoretically, λ_gas_ should be considered. These four groups of straw VIPs have the same core density and fixed panel volume, which means they have the same mass of straw core materials. Straw core materials consist of numerous straw fiber units so that the core materials made up of shorter fibers have more straw units, bringing higher porosity and smaller pore size. Consequently, λ_gas_ is lowered. 

## 4. Conclusions

VIPs, with the economic and environmentally friendly material straw as their core material, are new promising heat insulation materials which could sequester a lot of carbon if they were used on a large scale. Straw core has to be heated until it is strong enough. Here, FSP is an important turning point in the relationships between diametral compression strength-moisture and thermal conductivity-moisture content. The total thermal conductivity of straw VIP is sensitive to the diametral compression strength and moisture content of straw core materials. However, the length of straw is less important to the contribution of total thermal conductivity, although shorter fibers lead to relatively lower thermal conductivities. The optimum drying process for straw VIP is to heat the straw core at the temperature of 120 °C for 60 min, with its thermal conductivity of approximately 3.8 mW/(m·K). To be a potential candidate of VIP core materials, the relatively short service life of straw VIPs still needs to be studied in future research.

## Figures and Tables

**Table 1:** Comparison of VIPs prepared with different core material.

Core Material	Fiber Diameter (mm)	Pore Diameter (μm)	Porosity (%)	Bulk Density (kg/m^3^)	Thermal Conductivity mW/(m·K)
Straw	0.1–1.3 (mean 0.7)	2–50	>95%	100	3.5
PU [4]	--	150–200	>95%	55–65	≤10
PF [5]	--	200–400	98%	25	5
PS [6]	--	85–374	>93%	41–70	6.5

**Table 2:** Various properties at different drying temperature.

Drying Temperature (°C)	Moisture Content (%)	Diametral Compression Strength (MPa)	Thermal Conductivity mW/(m·K)
Ambient temp	5.62	0.24	37.0
	5.64	0.21	39.2
	5.62	0.25	36.7
	5.61	0.27	33.9
	5.63	0.23	38.2
60	0.34	0.29	13.4
	0.34	0.31	12.2
	0.35	0.25	14.3
	0.36	0.21	15.4
	0.32	0.37	10.2
90	0.13	0.29	7.6
	0.15	0.23	9.8
	0.13	0.29	7.7
	0.12	0.32	6.4
	0.12	0.32	6.2
120	0.08	0.53	3.8
	0.08	0.53	3.6
	0.09	0.48	4.0
	0.09	0.50	4.3
	0.08	0.55	3.5
150	0.04	0.08	28.0
	0.04	0.09	27.2
	0.03	0.07	29.5
	0.04	0.08	28.3
	0.05	0.12	26.1
180	0.01	0.06	32.1
	0.02	0.09	30.3
	0.02	0.09	30.0
	0.01	0.07	31.0
	0.01	0.08	30.4

Moisture content and diametral compression strength of straw core materials were measured immediately when VIP samples were prepared. Thermal conductivity was measured 3 days after sealing.

**Table 3:** Vacuum level within the VIP envelope enclosure changing with time.

Drying Temperature (°C)	Time (Day)	Inner Pressure (Pa)		Thermal Conductivity (mW/(m·K))	
		A	B	A	B
Ambient temp	3	5.6	5.1	37.0	36.7
	7	9.3	6.2	40.0	38.4
	30	16.8	10.5	45.7	41.2
60	3	5.3	4.5	13.1	12.9
	7	8.9	6.7	16.3	14.3
	30	16.2	9.8	24.6	18.1
90	3	5.1	4.2	7.6	7.4
	7	8.1	6.3	9.8	8.2
	30	15.9	9.3	17.3	12.0
120	3	4.6	3.9	3.9	3.8
	7	7.8	6.1	5.2	4.1
	30	15.3	8.7	12.9	7.7
150	3	4.5	3.9	28.0	27.6
	7	7.7	6.0	31.5	28.2
	30	15.2	8.6	40.2	32.5
180	3	4.4	3.8	31.0	30.3
	7	7.7	6.0	33.7	31.9
	30	15.2	8.7	44.3	36.2

A—VIP with 12 g of CaO powder desiccant and 2 g of wheat husks ash getter. B—VIP with 8 g of CaO powder desiccant and 6 g of wheat husks ash getter.
